# Molecular epidemiology and antimicrobial resistance profiles of *Klebsiella pneumoniae* isolates from hospitalized patients in different regions of China

**DOI:** 10.3389/fcimb.2024.1380678

**Published:** 2024-05-16

**Authors:** Yan Li, Chonghong Xie, Zhijie Zhang, Jianhua Liu, Hui Chang, Yong Liu, Xiaosong Qin

**Affiliations:** ^1^ Department of Laboratory Medicine, Shengjing Hospital of China Medical University, Shenyang, China; ^2^ Liaoning Clinical Research Center for Laboratory Medicine, Shenyang, China; ^3^ Center of Clinical Laboratory and Quality Control, Health Service Center of Liaoning Province, Shenyang, China

**Keywords:** *Klebsiella pneumoniae*, antimicrobial resistance, carbapenem-resistant, epidemiology, sequence type

## Abstract

**Introduction:**

The increasing incidence of Klebsiella pneumoniae and carbapenem-resistant Klebsiella pneumoniae (CRKP) has posed great challenges for the clinical anti-infective treatment. Here, we describe the molecular epidemiology and antimicrobial resistance profiles of K. pneumoniae and CRKP isolates from hospitalized patients in different regions of China.

**Methods:**

A total of 219 K. pneumoniae isolates from 26 hospitals in 19 provinces of China were collected during 2019–2020. Antimicrobial susceptibility tests, multilocus sequence typing were performed, antimicrobial resistance genes were detected by polymerase chain reaction (PCR). Antimicrobial resistance profiles were compared between different groups.

**Results:**

The resistance rates of K. pneumoniae isolates to imipenem, meropenem, and ertapenem were 20.1%, 20.1%, and 22.4%, respectively. A total of 45 CRKP isolates were identified. There was a significant difference in antimicrobial resistance between 45 CRKP and 174 carbapenem-sensitive Klebsiella pneumoniae (CSKP) strains, and the CRKP isolates were characterized by the multiple-drug resistance phenotype.There were regional differences among antimicrobial resistance rates of K. pneumoniae to cefazolin, chloramphenicol, and sulfamethoxazole,which were lower in the northwest than those in north and south of China.The mostcommon sequence type (ST) was ST11 (66.7% of the strains). In addition, we detected 13 other STs. There were differences between ST11 and non-ST11 isolates in the resistance rate to amikacin, gentamicin, latamoxef, ciprofloxacin, levofloxacin, aztreonam, nitrofurantoin, fosfomycin, and ceftazidime/avibactam. In terms of molecular resistance mechanisms, the majority of the CRKP strains (71.1%, 32/45) harbored blaKPC-2, followed by blaNDM (22.2%, 10/45). Strains harboring blaKPC or blaNDM genes showed different sensitivities to some antibiotics.

**Conclusion:**

Our analysis emphasizes the importance of surveilling carbapenem-resistant determinants and analyzing their molecular characteristics for better management of antimicrobial agents in clinical use.

## 1 Introduction

*Klebsiella pneumoniae* is a gram-negative bacterium capable of colonizing, invading, and causing infections in different anatomical sites of the human body. It is an opportunistic pathogen that can cause pneumonia, urinary tract infections, and bloodstream infections (Martin and Bachman, 2018). In China, the isolation rate of *K. pneumoniae* was second only to that of *Escherichia coli*, which ranks it second among gram-negative bacteria infections (Hu et al., 2019). With the excessive use and misuse of broad-spectrum antimicrobial agents, the increasing incidence of antimicrobial resistance has become a serious threat to public and global health as it complicates the clinical management of infectious diseases (Logan and Weinstein, 2017; van Duin and Doi, 2017; Liu et al., 2022). Thus, the World Health Organization (WHO) lists *K. pneumoniae* as one of the species of high priority and promotes the research and development of new antibiotics due to the growing global problem of antimicrobial resistance (Tacconelli et al., 2018).

Carbapenem antibiotics are still the “mainstay” in the treatment of *K. pneumoniae* infection at present. However, many carbapenem-resistant *Klebsiella pneumoniae* (CRKP) phenotypes have widely emerged in recent years. Yigit et al. (2001) first discovered CRKP in a hospital in North Carolina, USA, and subsequently, CRKP was reported in various countries (Mouloudi et al., 2014; Jin et al., 2015; Yu et al., 2016). The threat of drug-resistant bacterial infection posed by CRKP has garnered increasing attention worldwide. It poses a serious challenge to clinical management due to the limited availability of effective antimicrobial agents. Therefore, strengthening drug resistance monitoring and epidemiological research of CRKP is crucial to control the spread of multi-drug resistant bacteria and guide clinical rational anti-infection treatment.

The resistance mechanisms of CRKP mainly include the production of β-lactamases, overexpression of efflux pump genes, loss of outer membrane porins, and modification of drug targets. The production of carbapenemases is one of the major drivers of carbapenem resistance. The carbapenemase genes in CRKP strains mainly include *bla*_KPC_, *bla*_NDM_, *bla*_OXA_, and *bla*_IMP_, of which *bla*_KPC_ is the most clinically frequent in most countries (Tzouvelekis et al., 2012; Munoz-Price et al., 2013). To better understand CRKP epidemiology, explore the molecular resistance mechanisms, and analyze the characteristics of drug resistance, we initiated a multicenter surveillance study involving 26 hospitals in 19 provinces of China during 2019–2020.

In this study, we tested the resistance profiles of *K. pneumoniae* against 24 commonly used clinical antibiotics and compared the resistance rates among different regions to analyze the drug resistance status of *K. pneumoniae* in China. Using multilocus sequence typing (MLST), carbapenemase-resistant genes were detected in 45 CRKP strains and antimicrobial resistance among the different types was characterized, which provides experimental evidence for the effective treatment of CRKP infectious diseases.

## 2 Materials and methods

### 2.1 Collection and identification of strains

A total of 219 *K. pneumoniae* isolates were collected from 26 hospitals in China through the National Science and Technology Basic Research Program of China (2019FY101200). The participating hospitals are listed in **Table 1**. In October 2020, a retrospective collection of *K. pneumoniae* isolates from the participating facilities, collected between January 1 and December 31, 2019, was submitted to the central research team for further analysis. The strains originated from different sites of infection, including the respiratory tract, blood, enterocele, urine, skin and soft-tissue, central nervous system, reproductive tract, and digestive tract. The Qinling Mountains-Huaihe River line was used as the dividing line between the northern and southern regions; the Greater Hinggan Mountains-Yinshan-Helan Mountains line was used as the dividing line between the northern region and the northwestern region. All strains were identified using the VITEK-MS mass spectrometer (bioMerieux, France).

**Table 1:** Regional distribution of 219 *K. pneumoniae* isolates.

Regional distribution	Included hospitals	Numbers of isolates (%)
North	China-Japan Union Hospital of Jilin University Shengjing Hospital of China Medical University The Sixth People’s Hospital of Shenyang Peking Union Medical College Hospital Hebei Yanda Medical Research Institute Henan Provincial People’s Hospital General Hospital of Tianjin Medical University Beijing You’an Hospital Children’s Hospital of Soochow University Caohu People’s Hospital of Xiangcheng District	83 (37.9%)
South	The First Affiliated Hospital of Guangzhou Medical University The First Affiliated Hospital of Guangxi Medical University Southern University of Science and Technology Hospital Shenzhen Baoan District Maternal and Child Health Care Hospital Second Affiliated Hospital of Nanchang University Tongji Hospital of Huazhong University of Science and Technology Xiangya Hospital of Central South University First Affiliated Hospital of Kungming Medical University West China Hospital of Sichuan University Yunnan Cancer Hospital	85 (38.8%)
Northwest	No. 3201 Hospital General Hospital of Ningxia Medical University Tacheng District People’s Hospital People’s Hospital of Ningxia Hui Autonomous Region Dingxi People’s Hospital The Affiliated Hospital of Inner Mongolia Medical University	51 (23.3%)

### 2.2 Antimicrobial susceptibility testing

The antimicrobial susceptibility of the 219 *K. pneumoniae* strains was determined using the automated microorganisms drug sensitivity analyzer SAST60 (Deere, Zhuhai, China). Strains were identified as CRKP based on susceptibility testing against imipenem, meropenem, and ertapenem based on the minimum inhibitory concentrations (MIC) of ≥ 4 μg/mL for imipenem and meropenem, or ≥ 2 μg/mL for ertapenem as per the Clinical and Laboratory Standards Institute (CLSI) guidelines (CLSI M100-S33, 2023). ATCC 25922 was used as the quality control strain.

### 2.3 Multilocus sequence typing

MLST was performed as described on the Institute Pasteur database (http://bigsdb.pasteur.fr/klebsiella/). To identify the sequence types (STs) of the 45 CRKP strains, seven housekeeping genes, namely, *gapA*, *infB*, *mdh*, *pgi*, *phoE*, *rpoB*, and *tonB*, were amplified by polymerase chain reaction (PCR) and sequenced. The FASTA sequences of all these housekeeping genes were submitted to the *K. pneumoniae* MLST database, and the STs were determined.

### 2.4 PCR detection of antimicrobial resistance genes

The genomic DNA of the 45 CRKP isolates was extracted using a boiling method. Briefly, pure colonies that were cultured overnight on Columbia blood agar plates were suspended in sterile distilled water and the tubes placed in boiling water for 10 min, followed by rapid cooling in ice water. The lysate was then centrifuged at 10000 rpm for 2 min and the supernatant transferred to a sterile tube for further use. Six carbapenemase genes (*bla*_KPC_, *bla*_NDM_, *bla*_VIM_, *bla*_IMP_, *bla*_OXA-23_, and *bla*_OXA-48_) and extended spectrum β-lactamase genes (*bla*_CTX-M_) were amplified (Zhang et al., 2022). The detailed primer information and amplification conditions are listed in **Table 2**. Amplified products were visualized using 1% agarose gel electrophoresis. The amplified gene fragments were sequenced and alignments were performed using the Basic Local Alignment Search Tool (BLAST).

**Table 2:** Primers and amplification conditions of seven antimicrobial resistance genes.

Primer	Sequence	Sequence length (bp)	Amplification condition
*bla*_KPC_	(F)5’-ATGTCACTGTATCGCCGTCTA-3′ (R)5’-TTACTGCCCSKTGACGCCCAA-3′	822	94°C for 5 min, 30×(94°C for 60s, 55°C for 45s, and 72°C for 60s), 72°C for 5 min
*bla*_NDM_	(F)5’-GGTCGCGAAGCTGAGCACCGCAT-3′ (R)5’-GCAGCTTGTCGGCCATGCGGGC-3′	782	94°C for 4 min, 30×(94°C for 30s, 71°C for 30s, and 72°C for 50 s), 72°C for 10 min
*bla*_IMP_	(F)5’-GARGGYGTTTATGTTCATAC-3′ (R)5’-GTAMGTTTCAAGAGTGATRC-3′	587	94°C for 5 min, 30×(94°C for 60s, 55°C for 45s, and 72°C for 60 s), 72°C for 5 min
*bla*_VIM_	(F)5’-GTTTGGTCGCATATCKCAAC-3′ (R)5’-AATGCGCAGCACCAGGATAG-3′	382	94°C for 4 min, 30×(94°C for 30s, 58°C for 30s, and 72°C for 40 s), 72°C for 10 min
*bla*_OXA-48_	(F)5’-TTGGTGGCATCGATTATCGG-3’ (R)5’-GAGCACTTCTTTTGTGATGGC-3’	744	95°C for 5 min, 30×(94°C for 60s, 62°C for 60s, and 72°C for 60s), 72°C for 5 min
*bla*_OXA-23_	(F)5’-TGTACGGTTCAGCATAATTTA-3’ (R)5’-AGATGCCGGCATTTCTGACCG-3’	699	95°C for 5 min, 30×(94°C for 60s, 62°C for 60s, and 72°C for 60s), 72°C for 5 min
*bla*_CTX-M_	(F)5’-ATGTGCAGYACCAGTAARGTKATGGC-3’ (R)5’-TGGGTRAARTARGTSACCAGAAYCAGCGG-3’	593	94°C for 3 min, 25×(94°C for 60 s, 60°C for 60s, and 72°C for 60 s), 72°C for 10 min

### 2.5 Statistical analysis

Statistical analysis was performed using the 20.0 version of SPSS software. Categorical variables were described as proportions and associations assessed by Chi-square test or Fisher’s exact test. Results with *P-*values < 0.05 were considered statistically significant.

## 3 Results

### 3.1 Clinical characteristics associated with *K. pneumoniae* isolates

A total of 219 *K. pneumoniae* isolates were included in this study which originated from multiple sites of infection, including 22.4% (49/219) from the respiratory tract, 20.1% (44/219) from the blood, 16.4% (36/219) from enterocele, 13.7% (30/219) from urine, 12.8% (28/219) from skin and soft tissue, 7.8% (17/219) from the central nervous system, 5.0% (11/219) from the reproductive tract, and 1.8% (4/219) from the digestive tract. Of all the enrolled patients, 60.9% (134/219) were male, and the ages of all patients ranged from 1 day to 95 years old (median age: 54 years old). The proportions of *K. pneumoniae* isolates from patients in the surgical wards, medical wards, and intensive care units (ICUs) were 32.3%, 25.5%, and 15.5%, respectively.

### 3.2 Antimicrobial susceptibility

The antimicrobial resistance rates of the *K. pneumoniae* isolates are shown in **Table 3**. The resistance rates of *K. pneumoniae* isolates to imipenem, meropenem, and ertapenem were 20.1%, 20.1%, and 22.4%, respectively. Strains resistant to at least one of the three antimicrobial agents were identified as CRKP, which accounted for 20.5% (45/219) of all strains.

**Table 3:** Antimicrobial susceptibility of the 219 *Klebsiella pneumoniae* isolates to 24 antimicrobial agents.

Antimicrobial Agent	Antibiotic susceptibility (%)			MIC (μg/mL) (CLSI,2023)		
	S	I	R	S	I	R
Amoxicillin/clavulanic acid	60.3	8.7	31.0	≤8/4	16/8	≥32/16
Ampicillin/Sulbactam	54.3	11.0	34.7	≤8/4	16/8	≥32/16
Piperacillin/Tazobactam	76.7	1.4	21.9	≤8/4	16/4	≥32/4
Amikacin	84.5	0	15.5	≤4	8	≥16
Gentamicin	71.7	0.9	27.4	≤2	4	≥8
Ceftazidime	69.8	1.4	28.8	≤4	8	≥16
Ceftriaxone	58.5	0.9	40.6	≤1	2	≥4
Cefuroxime	18.7	38.8	42.5	≤4	8–16	≥32
Ceftazidime/Avibactam	93.6	0	6.4	≤8/4	–	≥16/4
Cefazolin	50.3	5.0	44.7	≤2	4	≥8
Cefoxitin	69.0	2.7	28.3	≤8	16	≥32
Latamoxef	76.7	2.3	21.0	≤8	16–32	≥64
Ciprofloxacin	52.0	12.8	35.2	≤0.25	0.5	≥1
Levofloxacin	57.1	12.3	30.6	≤0.5	1	≥2
Imipenem	79.0	0.9	20.1	≤1	2	≥4
Meropenem	78.5	1.4	20.1	≤1	2	≥4
Ertapenem	77.2	0.4	22.4	≤0.5	1	≥2
Aztreonam	66.7	3.7	29.6	≤4	8	≥16
Chloramphenicol	65.3	5.5	29.2	≤8	16	≥32
Colistin	24.2	72.1	3.7	–	≤2	≥4
Nitrofurantoin	48.8	28.8	22.4	≤32	64	≥128
Fosfomycin	84.5	5.9	9.6	≤64	128	≥256
Minocycline	75.3	14.2	10.5	≤4	8	≥16
Sulfamethoxazole	60.7	0	39.3	≤2/38	8	≥4/76

S, susceptible; R, resistant; I, intermediate.

The character "-" indicates that there is no explanation for the break point.

Then, we divided the regional source of strains into three groups: north, south, and northwest according to geographical region, and compared the difference in the antimicrobial resistance rates among these regions by Chi-square test; *P* < 0.05 indicated that there were differences among the groups. Statistical results showed that some of the antimicrobial resistance rates were different in different regions of China. For example, the resistance rates of cefazolin, chloramphenicol, and sulfamethoxazole in northwest were lower than those in north and south of China (**Table 4**).

**Table 4:** Antimicrobial resistance profiles of 219 *K. pneumoniae* isolates from different regions in China.

Antimicrobial Agent	North (N=83)	South (N=85)	Northwest (N=51)	χ2	*P* value
Amoxicillin/clavulanic acid	34 (41.0%)	37 (43.5%)	16 (31.4%)	2.053	0.358
Ampicillin/Sulbactam	42 (50.6%)	41 (48.2%)	17 (33.3%)	4.168	0.124
Piperacillin/Tazobactam	23 (27.7%)	20 (23.5%)	8 (15.7%)	2.561	0.278
Amikacin	14 (16.9%)	14 (16.5%)	6 (11.8%)	0.722	0.697
Gentamicin	25 (30.1%)	25 (29.4%)	12 (23.5%)	0.759	0.684
Ceftazidime	30 (36.1%)	26 (30.6%)	10 (19.6%)	4.116	0.128
Ceftriaxone	36 (43.4%)	41 (48.2%)	14 (27.5%)	5.852	0.054
Cefuroxime	69 (83.1%)	70 (82.4%)	39 (76.5%)	1.027	0.598
Ceftazidime/Avibactam	7 (8.4%)	3 (3.5%)	4 (7.8%)	1.922	0.383
Cefazolin	44 (53.0%)	49 (57.6%)	16 (31.4%)	9.364	**0.009**
Cefoxitin	31 (37.3%)	27 (31.8%)	10 (19.6%)	4.678	0.096
Latamoxef	22 (26.5%)	21 (24.7%)	8 (15.7%)	2.226	0.328
Ciprofloxacin	43 (51.8%)	45 (52.9%)	17 (33.3%)	5.709	0.058
Levofloxacin	37 (44.6%)	40 (47.1%)	17 (33.3%)	2.601	0.272
Imipenem	21 (25.3%)	18 (21.2%)	7 (13.7%)	2.554	0.279
Meropenem	21 (25.3%)	19 (22.4%)	7 (13.7%)	2.577	0.276
Ertapenem	23 (27.7%)	20 (23.5%)	7 (13.7%)	3.545	0.170
Aztreonam	27 (32.5%)	34 (40.0%)	12 (23.5%)	3.930	0.140
Chloramphenicol	34 (41.0%)	32 (37.6%)	10 (19.6%)	6.889	**0.032**
Colistin	3 (3.6%)	4 (4.7%)	1 (2.0%)	0.683	0.711
Nitrofurantoin	42 (50.6%)	44 (51.8%)	26 (51.0%)	0.023	0.988
Fosfomycin	15 (18.1%)	14 (16.5%)	5 (9.8%)	1.741	0.419
Minocycline	25 (30.1%)	28 (32.9%)	14 (27.5%)	0.467	0.792
Sulfamethoxazole	35 (42.2%)	39 (45.9%)	12 (23.5%)	7.149	**0.028**

P<0.05 was considered to be statistically significant.

Bold characters indicate a *P* value less than 0.05.

Moreover, CRKP exhibited 100% resistance rate to ampicillin/sulbactam, ceftazidime, ceftriaxone, cefuroxime, cefazolin, cefoxitin, and ertapenem. The antimicrobial resistance rates of CRKP isolates to all tested antibiotics except for polymyxin were significantly different to the CSKP (*P <*0.05) (**Table 5**).

**Table 5:** Comparison of antimicrobial resistance rates between CRKP and CSKP clinical isolates.

Antimicrobial Agent	CRKP(N=45)		CSKP(N=174)		χ2	*P* value
	IR(n)	IR(%)	IR(n)	IR(%)		
Amoxicillin/clavulanic acid	44	97.8	24	13.8	85.933	0.000
Ampicillin/Sulbactam	45	100	31	17.8	67.399	0.000
Piperacillin/Tazobactam	43	95.6	5	2.9	175.919	0.000
Amikacin	29	64.4	5	2.9	103.349	0.000
Gentamicin	31	68.9	29	16.7	45.951	0.000
Ceftazidime	45	100	18	10.3	131.297	0.000
Ceftriaxone	45	100	44	25.3	79.667	0.000
Cefuroxime	45	100	48	27.6	13.046	0.000
Ceftazidime/Avibactam	34	75.6	3	1.7	138.808	0.000
Cefazolin	45	100	53	30.5	57.158	0.000
Cefoxitin	45	100	17	9.8	125.770	0.000
Latamoxef	41	91.1	5	2.9	186.572	0.000
Ciprofloxacin	39	86.7	38	21.8	46.750	0.000
Levofloxacin	37	82.2	30	17.2	39.858	0.000
Imipenem	40	88.9	0	0.0	200.936	0.000
Meropenem	41	91.1	0	0.0	212.910	0.000
Ertapenem	45	100	0	0.0	213.008	0.000
Aztreonam	41	91.1	24	13.8	85.083	0.000
Chloramphenicol	23	51.1	41	23.6	25.536	0.000
Colistin	2	4.4	6	3.4	0.121	0.728
Nitrofurantoin	35	77.8	14	8.0	28.607	0.000
Fosfomycin	19	42.2	2	1.1	54.689	0.000
Minocycline	7	15.6	16	9.2	6.452	0.011
Sulfamethoxazole	31	68.9	55	31.6	20.835	0.000

IR, intermediate and resistant; P<0.05 was considered to be statistically significant.

### 3.3 MLST analysis of CRKP isolates

To further understand the genetic diversity of CRKP, we performed MLST analysis (**Table 6**). The results showed that 14 distinct STs were identified within the 45 isolates, and the diversity index was 31.1% (14/45). Among them, the most frequently represented ST was ST11 (66.7%,30/45). Other STs included ST48 (4.4%,2/45) and ST299 (4.4%,2/45), and ST23, ST2807, ST15, ST147, ST3410, ST4926, ST661, ST367, ST638, ST345, and ST1540 accounted for 2.2% of the isolates.

**Table 6:** Antimicrobial resistance of 45 CRKP isolates against antimicrobial agents among different STs.

Antimicrobial Agent	ST11(N=30)		non-ST11(N=15)		χ2	*P* value
	IR(n)	IR(%)	IR(n)	IR(%)		
Amoxicillin/clavulanic acid	30	100	14	93.3	2.045	0.153
Ampicillin/Sulbactam	30	100	15	100	—	—
Piperacillin/Tazobactam	29	96.7	14	93.3	0.262	0.609
Amikacin	24	80.0	5	33.3	9.504	0.002
Gentamicin	25	83.3	6	40.0	8.762	0.003
Ceftazidime	30	100	15	100	—	—
Ceftriaxone	30	100	15	100	—	—
Cefuroxime	30	100	15	100	—	—
Ceftazidime/Avibactam	0	0	11	73.3	29.118	0.000
Cefazolin	30	100	15	100	—	—
Cefoxitin	30	100	15	100	—	—
Latamoxef	30	100	11	73.3	8.780	0.003
Ciprofloxacin	30	100	9	60.0	13.846	0.000
Levofloxacin	30	100	7	46.7	19.459	0.000
Imipenem	29	96.7	11	73.3	5.513	0.019
Meropenem	29	96.7	12	80.0	3.430	0.064
Ertapenem	30	100	15	100	—	—
Aztreonam	30	100	11	73.3	8.780	0.003
Chloramphenicol	18	60.0	5	33.3	2.846	0.092
Colistin	2	6.7	0	0	1.047	0.306
Nitrofurantoin	30	100	5	33.3	25.714	0.000
Fosfomycin	17	56.7	2	13.3	7.697	0.006
Minocycline	3	10.0	4	26.7	2.115	0.146
Sulfamethoxazole	19	63.3	12	80.0	1.296	0.255

IR, intermediate and resistant; P<0.05 is considered to be statistically significant.

The character "—" indicates that it cannot be compared between these two groups.

### 3.4 Mechanisms of carbapenemase genes among clinical CRKP strains

The majority of CRKP (71.1%, 32/45) were found to harbor *bla*_KPC-2_, while the rest carried *bla*_NDM_(22.2%, 10/45), *bla*_CTX-M_(4.4%, 2/45), and *bla*_OXA-23_ (2.2%, 1/45). Other carbapenemase genes, including *bla*_OXA-48_, *bla*_VIM_, and *bla*_IMP_ were not detected in these strains. Both *bla*_KPC_ and *bla*_NDM_ were the most commonly identified in this study. Strains carrying different carbapenemases showed different sensitivity to antibiotics (**Table 7**). Most CRKP strains with *bla*_KPC_ were resistant to ciprofloxacin, levofloxacin, nitrofurantoin, fosfomycin, chloramphenicol, and aztreonam, whereas CRKP strains with *bla*_NDM_ were susceptible to these antibiotics. There were significant differences in resistance to ceftazidime/avibactam between *bla*_KPC_ and *bla*_NDM_. Only one CRKP strain with *bla*_KPC_ was resistant to ceftazidime/avibactam, while almost all CRKP strains with *bla*_NDM_ were resistant to the antibiotic.

**Table 7:** Comparison of antibiotic resistance profiles of 45 CRKP isolates harboring *bla_KPC_* and *bla_NDM_*.

Antimicrobial Agent	*bla*_KPC_ (N=32)	*bla*_NDM_ (N=10)	χ2	*P* value
Amoxicillin/clavulanic acid	32 (100%)	10 (100%)	—	—
Ampicillin/Sulbactam	32 (100%)	10 (100%)	—	—
Piperacillin/Tazobactam	31 (96.9%)	10 (100%)	0.000	1.000
Amikacin	24 (75%)	4 (40%)	2.773	0.096
Gentamicin	25 (78.1%)	4 (40%)	3.551	0.059
Ceftazidime	32 (100%)	10 (100%)	—	—
Ceftriaxone	32 (100%)	10 (100%)	—	—
Cefuroxime	32 (100%)	10 (100%)	—	—
Ceftazidime/Avibactam	1 (3.1%)	10 (100%)	32.147	0.000
Cefazolin	32 (100%)	10 (100%)	—	—
Cefoxitin	32 (100%)	10 (100%)	—	—
Latamoxef	31 (96.9%)	10 (100%)	0.000	1.000
Ciprofloxacin	32 (100%)	5 (50%)	13.707	0.000
Levofloxacin	32 (100%)	3 (30%)	22.076	0.000
Imipenem	31 (96.9%)	9 (90%)	0.002	0.968
Meropenem	31 (96.9%)	10 (100%)	0.000	1.000
Ertapenem	32 (100%)	10 (100%)	—	—
Aztreonam	32 (100%)	6 (60%)	9.886	0.002
Chloramphenicol	19 (59.4%)	2 (20%)	4.725	0.030
Colistin	2 (6.3%)	0	0.000	1.000
Nitrofurantoin	30 (93.8%)	2 (20%)	18.959	0.000
Fosfomycin	17 (53.1%)	1 (10%)	4.159	0.041
Minocycline	4 (12.5%)	1 (10%)	0.000	1.000
Sulfamethoxazole	21 (65.6%)	7 (70%)	0.000	1.000

P < 0.05 was considered to be statistically significant.

## 4 Discussion

In this study, we collected 219 *K. pneumoniae* isolates from different regions of China in 2020. The main sources of specimens were surgical wards (32.4%), medical wards (25.4%), and ICUs (15.5%). This may be related to more invasive procedures such as ventilator placement and indwelling devices in surgical wards and ICUs, as well as longer hospital stay and long-term use of antibiotics (Karakonstantis et al., 2020). Tian et al. (2016) also reported that morbidity and mortality rates for CRKP-infected patients in ICUs were much higher than those for non-ICU patients. This suggests that environmental hygiene and disinfection of medical devices should be strengthened in clinical settings, and attention should be paid to aseptic practices of medical staff as well as the rational use of antibiotics.

We surveyed the molecular characteristics and antibiotic resistance profiles of our *K. pneumoniae* isolates. The results showed that *K. pneumoniae* is inherently resistant to ampicillin, and the resistance rates of the first, second, and third generation cephalosporins exceeded 40%. This implied that cephalosporins in China may be excessively used. Carbapenem antibiotics are the last line of defense against *K. pneumoniae*; however, with the widespread use of these antibiotics, the detection rates of CRKP strains had been increasing gradually. In our study, among the 219 *K. pneumoniae* isolates, 45 (20.5%) were identified as CRKP. This is generally consistent with other research. For example, Hu et al. (2019) reported the resistance rates of *K. pneumoniae* to imipenem and meropenem as 25% and 26.3%, respectively, in 2018. In addition, a retrospective observational study from Zhejiang, China suggested that imipenem and meropenem resistance rates in 2018 were 15.8% and 20.9%, respectively (Hu et al., 2020), which were lower than the rates observed in our study. Notably, most CRKP strains showed multiple drug resistance; thus, we compared the difference of antibiotics resistance between CRKP and CSKP. Our findings confirmed that CRKP strains were resistant to most of clinically common antibiotics, which included penicillins, cephalosporins, aminoglycosides, quinolones, carbapenems, and so on, but not for colistin and minocycline.

Antimicrobial Resistance Collaborators (2022) estimated that Australasia had the lowest antimicrobial resistance burden among the 21 Global Burden of Diseases, Injuries, and Risk Factors Study regions in 2019, while the highest rates of antimicrobial resistance burden were in sub-Saharan Africa. This confirmed that bacterial antimicrobial resistance rates differed by geographic location. China as a vast nation exhibited significant variations in the levels of antibiotic resistance across different provinces and regions. In our study, there were regional differences among antimicrobial resistance rates of *K. pneumoniae* to cefazolin, chloramphenicol, and sulfamethoxazole. Resistance to these antibiotics was lower in Northwest China, but there was no difference in resistance to other antibiotics. This difference may hint at differences in medication habits in different regions. Because of these regional differences, the epidemiological precautions and medication policies that guide them also differ geographically. In recent years, China has issued the “National Action Plans for Combating Bacterial Resistance (2022–2025)”, implementing effective antimicrobial stewardship and developing a rational antibiotic use policies (Ding and Hu, 2023). China’s efforts to combat antimicrobial resistance have shown significant results. According to CHINET, the resistance rates of *K. pneumoniae* to imipenem and meropenem have straightly climbed from 3.0% and 2.9% in 2005, to 25.0% and 26.3% in 2018 (Hu et al., 2019). However, a stabilizing trend is observed from 2019 onwards, with rates of 22.6% and 24.2% in 2022 (Qin et al., 2024). Therefore, drug use in various regions should be adapted to local conditions through the use of corresponding drugs according to the regional resistance rate to replace other antibiotics. For example, in areas with high rates of chloramphenicol resistance, use of chloramphenicol should be reduced. Therefore, it is necessary to continuously monitor the molecular epidemiological characteristics of the same strain isolate in a specific region, so as to develop appropriate antibiotic therapy and reduce unnecessary antibiotic treatment.

MLST of 45 CRKP isolates was conducted, and a total of 14 ST types were identified, of which, ST11 accounted for the largest proportion (about 66.7%). There are 13 other ST types, accounting for a smaller proportion, including ST48, ST299, ST23, ST2807, ST15, ST147, ST3410, ST4926, ST661, ST367, ST638, ST345, and ST1540. Although the number of the strains included in this study was insufficient, it still showed that the distribution of ST had strong regional characteristics. At present, there are more than 2000 *K. pneumoniae* STs identified globally. The predominant ST among CRKP strains in European countries, such as Norway and Sweden, and in the United States, is ST258 (Deleo et al., 2014), whereas ST11 predominates in China (Zhang et al., 2017; Zhou et al., 2020). Liu et al. (2022) reported that ST11 accounted for 64.2% of all CRKP strains in China in 2016–2020, which was consistent with our results, showing that ST11 was the most common type of CRKP strains. The number of virulence plasmids and mobile elements carried by ST11 were higher than those of other ST strains, which resulted in the dissemination of virulence plasmids in hospitals and different areas (Mathers et al., 2011; Martin et al., 2017). Horizontal gene transfer within and between bacterial species ultimately led to the high prevalence of ST11 in China.

Carbapenem antibiotics are a general term for a class of broad-acting antibiotics that are commonly used as last-resort antibiotics to treat serious bacterial infections. Therefore, we focused on the resistance of carbapenem antibiotics and deeply researched carbapenemase as it proved to be the main mechanism of drug resistance in CRKP. In our study, 71.1% (32/45) of CRKP isolates were found to harbor *bla*_KPC-2_, followed by *bla*_NDM_ (22.2%, 10/45), *bla*_CTX-M_ (4.4%, 2/45), and *bla*_OXA-23_ (2.2%, 1/45). Among 32 strains harboring *bla*_KPC_, 93.8% (30/32) belonged to ST11, whereas the STs of the strains carrying *bla*_NDM_ were more dispersed. *K. pneumoniae* carbapenemase (KPC)-producing CRKP was reported first in 2007 (Wei et al., 2007), and *bla*_KPC-2_ subsequently became the dominant genotype in China (Zhang et al., 2018; Li et al., 2022), which was most commonly associated with the epidemic clone ST11 (Liu et al., 2018). KPC enzymes account for more than 70% of CRKP, followed by New Delhi metallo-beta-lactamase (NDM)-producing strains, while the prevalence of impenemase (IMP), verona integron-encoded metallo-beta-lactamase (VIM), and oxacillinase-48 (OXA-48) enzymes is relatively low in China (Hu et al., 2020), which was similar to our results.

We also discussed the relationship between antimicrobial resistance and different STs or carbapenemase genotypes. The results showed that antibiotic resistance is also different in different STs. The resistance rates of ST11 to amikacin, gentamicin, latamoxef, ciprofloxacin, levofloxacin, aztreonam, nitrofurantoin, and fosfomycin were higher than those of non-ST11. In contrast, ST11 was sensitive to ceftazidme/avibactam, while non-ST11 showed resistance to it. Of all the 45 CRKP isolates, there were 32 strains harboring *bla*_KPC_ and 10 strains carrying *bla*_NDM_. Antibiotic resistance analysis found that the resistance rates of the two strains to some antibiotics differed, and the difference was statistically significant. CRKP strains with *bla*_KPC_ showed higher rates of resistance to ciprofloxacin, levofloxacin, nitrofurantoin, fosfomycin, chloramphenicol, and aztreonam, while CRKP strains with *bla*_NDM_ were almost completely resistant to ceftazidime/avibactam. Similar to previous findings, *bla*_NDM_-carrying CRKP strains were resistant to ceftazidime/avibactam, but *bla*_KPC_-containing CRKP strains were sensitive to it. This indicated that CRKP resistance is closely related to its different resistance genotypes and STs. CRKP carrying *bla*_KPC_ and *bla*_NDM_ differed in antibiotic resistance, with different resistance to different antibiotics. The detection of drug resistance genotypes would be helpful to adjust the antibiotic application strategy in a timely manner.

This study has a few limitations. First, the number of strains included in this study may be insufficient to provide a comprehensive representation of the molecular epidemiological characteristics and antimicrobial resistance profiles of *K. pneumoniae* and CRKP in China. Second, whole genome sequencing of the CRKP was not performed, which would be included in our further research priorities to provide deeper insights into the pathogens.

## 5 Conclusions

This study described the prevalence and molecular epidemiological characteristics of *K. pneumoniae* and CRKP in China during 2019–2020. The resistance rates of *K. pneumoniae* to different antibiotics differed by region, which could be attributed to regional variations in antibiotic use. Compared with CSKP, CRKP had more severe drug resistance and showed more multiple drug resistance rates. Although CRKP exhibited a wide range of strain types, ST11 was still the major strain type. Other strain types were relatively rare and more sporadic. In terms of molecular resistance mechanisms, the majority of the CRKP strains harbored *bla_KPC-2_*, followed by *bla_NDM_*. Moreover, there was an association between antibiotic resistance profiles of CRKP strains with different STs and different carbapenemase genes. Hence, understanding the resistance phenotype and molecular epidemiology of CRKP isolates is essential to control the transmission of these drug-resistant bacteria and to promote the rational use of antibiotics.

## Data availability statement

The raw data supporting the conclusions of this article will be made available by the authors, without undue reservation.

## Ethics statement

In accordance with the local legislation and institutional requirements, ethical review and approval were waived for this study as the samples were collected during routine care. Patient consent was waived in accordance with the national legislation and the institutional requirements as the patient data were completely anonymized and study findings were not used for the clinical management of the patients.

## Author contributions

YaL: Formal analysis, Investigation, Methodology, Writing – original draft, Data curation. CX: Writing – review & editing, Formal analysis, Investigation, Methodology, Validation. ZZ: Writing – review & editing. JL: Writing – review & editing. HC: Data curation, Writing – review & editing. YoL: Writing – review & editing, Funding acquisition, Project administration, Resources. XQ: Conceptualization, Project administration, Supervision, Writing – review & editing.
